# 

**Published:** 1911-09

**Authors:** 


					﻿PUBLISHED MONTHLY.	ATL ANTA	SUBSCRIPTION $1.00
JOURNAL-RECOEOap
OF MEDIClfe”
Successor	^e^*ca’ an<^ Surgical Journal, Established 1855. / E74-L Vpnr
| and Southern Medical Record, Established 1870.	) 3/Lil I Cd I
Vol. LVII. Atlanta, Ga., September 1911.	No. 6
				

